# QiShenYiQi pill for myocardial collagen metabolism and apoptosis in rats of autoimmune cardiomyopathy

**DOI:** 10.1080/13880209.2022.2056206

**Published:** 2022-04-01

**Authors:** Shichao Lv, Wanqin Zhang, Peng Yuan, Chunmiao Lu, Jianping Dong, Junping Zhang

**Affiliations:** a First Teaching Hospital of Tianjin University of Traditional Chinese Medicine (National Clinical Research Center for Chinese Medicine Acupuncture and Moxibustion), Tianjin, China; b Tianjin Key Laboratory of Traditional Research of TCM Prescription and Syndrome, Tianjin, China; c Jiashan Hospital of Traditional Chinese Medicine, Zhejiang, China; d Health Center of Balitai Town, Jinnan, China

**Keywords:** Cardiac myosin, myocardial remodelling, cell apoptosis, traditional Chinese medicine

## Abstract

**Context:**

QiShenYiQi pill (QSYQ) is a traditional Chinese medicine with a myocardial protective effect.

**Objective:**

To explore the effect of QSYQ on myocardial collagen metabolism in rats with autoimmune cardiomyopathy and explore the underlying mechanism from the aspect of apoptosis.

**Materials and methods:**

We established an autoimmune cardiomyopathy model using Lewis rats. The rats were then randomly divided into six groups (n = 8): control, model, 3-methyladenine (15 mg/kg, intraperitoneal injection), QSYQ low-dose (135 mg/kg, gavage), QSYQ medium dose (270 mg/kg, gavage), and QSYQ high-dose (540 mg/kg, gavage) for four weeks. Van Gieson staining was applied for myocardial pathological characteristics, TUNEL fluorescence for myocardial cell apoptosis, enzyme-linked immunosorbent assay (ELISA) for serum PICP, PIIINP, and CTX-I levels, and western blot analysis for type I/III myocardial collagen, Bcl-2, Bax, and caspase-3 proteins.

**Results:**

Results showed that QSYQ (135, 270, or 540 mg/kg) significantly reduced the expression of myocardial type I/III collagen, and concentrations of serum PICP, PIIINP, and CTX-I in rats. Moreover, QSYQ could alleviate myocardial fibrosis more effectively at a higher dose. QSYQ could also inhibit myocardial apoptosis via downregulating Bcl-2 expression, and upregulating Bax and caspase-3 expression levels.

**Discussion and conclusions:**

The QSYQ can improve myocardial collagen metabolism by inhibiting apoptosis, which provides a potential therapeutic approach for autoimmune cardiomyopathy.

## Introduction

In the past few years, viral diseases have gradually increased largely due to the surge of new virulent viruses. About 5% of patients infected with cardiophilic viruses had heart involvement, and with the number going up to 10% in some regions (Lv et al. 2013). Coxsackievirus B3 (CVB3) and parvovirus 19 (PVB19) are responsible for the progression of myocarditis to dilated cardiomyopathy (DCM) (Flynn et al. 2017; Zhao and Fu 2018; Maisch and Pankuweit 2020). Myocarditis can be caused by several viruses, including influenza A (H1N1) virus (Sellers et al. 2017), Middle East respiratory syndrome (MERS) coronavirus (MERS-CoV) (Alhogbani 2016), and even the novel coronavirus (severe acute respiratory syndrome-coronavirus-2, SARS-CoV-2), which is responsible for the current global pandemic (Bavishi et al. 2020; Bansal 2020; Irabien-Ortiz et al. 2020; Doyen et al. 2020; Madjid et al. 2020). Previous studies have suggested that DCM can be divided into three stages: viral replication, immune activation, and cardiomyopathy (Liu and Mason 2001; Cannata' et al. 2019). It is worth noting that virus infection, myocarditis, and immunodeficiency are primary risk factors for DCM incidence, with the overactivation of the immune system following virus infection being the key factor (Donal et al. 2019).

In the early stage, the virus attacks the cardiac muscle, thereby leading to myocardial injury and dysfunction, followed by secondary autoimmune responses to induce progressive injury. Notably, cardiac myosin serves as the main target of antigens during the process, whereas virus infection can only be a predisposing factor for cardiomyopathy. For further progress from myocarditis to myocardial fibrosis and even progression to DCM, a persistent autoimmune response is the key part that cannot be negated (Bracamonte-Baran and Čiháková 2017; Akhtar and Elliott 2019). As the most prevalent chronic cardiomyopathy, DCM is a progressive disease characterised by high mortality, which was reported to be about 46% in five years at the Mayo Clinic (Grogan et al. 1995; Harvey and Leinwand 2011). DCM treatment involves improving myocardial remodelling, which helps patients to benefit from immunoadsorption therapy, a potential clinical application. Nevertheless, the appropriate population for immunotherapy is still unclear, and the evidence grading of evidence-based medicine needs to be further improved (Heymans et al. 2016; Chinese Society of Cardiology and Chinese myocarditis and cardiomyopathy cooperation group 2018; Bruestle et al. 2020).

Traditional Chinese medicine (TCM) can effectively target the immunomodulatory function of inflammatory factors, thereby treating the myocardial injury caused by abnormal immune responses in early DCM (Liu et al. 2018). QiShenYiQi pill (QSYQ), a traditional Chinese herbal medicine, is composed of *Astragalus membranaceus* (Fisch.) Bunge (Leguminosae), *Salvia miltiorrhiza* Bunge (Labiatae), *Panax pseudo-ginseng* Wall. var. *notoginseng* (Burkill) Hoo & Tseng (Araliaceae), and *Dalbergia odorifera* T. Chen (Leguminosae). Studies have shown that the main active ingredients of QSYQ are astragaloside, tanshinol, protocatechualdehyde, and ginsenoside Rg1 and Rb1 (Fu et al. 2012). It should be noted that QSYQ, produced by the Tasly Pharmaceutical Group Co., Ltd. (Tianjin, China), was approved by the China State Food and Drug Administration in 2003 for treating coronary heart disease and angina pectoris (Tang et al. 2013). According to the instructions, the specification of QSYQ is 500 mg/bag, and the commonly used clinical dose is one bag each time, three times a day. Several studies have demonstrated that QSYQ could prevent the high-glucose induced H9c2 myocardial injury (Zhang et al. 2019), suppress the overload-induced myocardial fibrosis (Chen et al. 2015; Ruan et al. 2018), and alleviate the myocardial ischemia-reperfusion (I/R) injury (Chen et al. 2015; Zheng et al. 2019).

In our previous study, we found that QSYQ could inhibit the hyperactive proliferation of cardiac fibroblasts (CFs) in rat models induced by transforming growth factor β1 (TGF-β1), which helped to improve the myocardial injury in rats with autoimmune myocarditis (Lv et al. 2016; Ma et al. 2017). However, the effect of QSYQ on myocardial collagen metabolism and apoptosis in rats with autoimmune cardiomyopathy is not yet fully understood. This study applied cardiac myosin injection in rats to induce autoimmune cardiomyopathy, with the overarching goal of exploring the efficacy of QSYQ against myocardial collagen metabolism and elucidating the underlying mechanism from the aspect of apoptosis.

## Materials and methods

### Experimental animals

Lewis male rats (body mass: 230 ± 20 g) were obtained from Beijing Vital River Laboratory Animal Technology Co., Ltd. (SCXK [Beijing] 2016-0011), and housed in the Institute of Radiation Medicine, Chinese Academy of Medical Sciences. Rats were allowed *ad libitum* access to food and water under an environment with a temperature of 20–24 °C, a relative humidity of 45–55%, and a 12 h light/dark cycle. The study was approved by the animal ethics committee of Tianjin University of Traditional Chinese Medicine (No. TCM-LAEC2016016), and was conducted in accordance with the Guide for the Care and Use of Laboratory Animals issued by National Institutes of Health (NIH) (No. 85-23, edited in 1996).

### Primary reagents

QiShenYiQi Pill (Z20030139) (Tasly Pharmaceutical Group Co., Ltd., Tianjin, China); cardiac myosin (M0531) and Freund's complete adjuvant (FCA) (F5881) (Sigma); 3-methyladenine (A8353) (ApexBio Technology); Van Gieson staining solution (DC0047) (Beijing Leagene Biotechnology Co., Ltd., Beijing, China); enzyme linked immunosorbent assay (ELISA) for procollagen type I carboxy-terminal propeptide (PICP) (CSB-E08081r), procollagen III N-terminal peptide (PIIINP) (CSB-E08096r) and collagen type I C-terminal peptide (CTX-I) (CSB-E12776r) (Cusabio Biotech Co., Ltd., Wuhan, China); phosphatase inhibitor (PI0015), RIPA lysis buffer (PS0012) and protease inhibitor mixture (PI0015) (Beijing Leagene Biotechnology Co., Ltd., Beijing, China); ECL chemiluminescence detection kit (B500022), anti-collagen I (14695-1-AP), anti-collagen III (22734-1AP), anti-Bcl-2 (12789-1-AP), anti-Bax (50599-2-Ig), anti-caspase3 (19677-1-AP), anti-β-actin and secondary horse radish peroxidase (HRP)-conjugated goat anti-rabbit antibody (SA00001-2) (Proteintech Group, USA); BCA protein assay kit (AR0146) (Wuhan Boster Biological Technology Co., Ltd., Wuhan, China); protease K (1245680100), and antifade mounting medium (S2110) (Beijing Solarbio Science & Technology Co., Ltd., Beijing, China); DAPI staining solution (C02-04002) (Beijing Biosynthesis Biotechnology Co., LTD., China); TUNEL kit (11684817910) (Roche).

### Model establishment and grouping

Cardiac myosin injection was applied to induce autoimmune cardiomyopathy in rats (Kodama et al. 1990). Rats were then divided into six groups (*n* = 8): control group (equal volume of distilled water by gavage), model group (equal volume of distilled water by gavage), 3-methyladenine group (15 mg/kg, intraperitoneal injection), QSYQ low-dose group (135 mg/kg by gavage), QSYQ medium-dose group (270 mg/kg by gavage), and QSYQ high-dose group (540 mg/kg by gavage). Porcine cardiac myosin (6.4 mg/mL) was mixed with FCA containing dried *Mycobacterium tuberculosis* (1 mg/mL) at a volume ratio of 1:1. The mixture was then administered (0.3 mL) via lower limb footpad injection on the 1^st^ and 7^th^ days in rats of all groups, with exception of rats in the control group which were administered with a mixture of phosphate buffered saline (PBS) and FCA (1:1) under the same conditions. Four weeks after the first injection, QSYQ solution was separately administered by gavage in the low-, medium-, and high-dose groups, whereas distilled water of equal volume was administered for the control and model groups. According to the convertion of body surface area, the dosage of the rats was 135 mg/kg in the low-dose group, the middle dose was two times the low dose, and the high dose was four times the low dose (Xu et al. 2002).

### Sample collection and processing

Samples were collected four weeks after drug intervention. Briefly, rats in each group were weighed before sampling, followed by collection of blood samples from the abdominal aorta instantly after anaesthesia by intraperitoneal injection of 3% pentobarbital sodium (45 mg/kg). Next, the blood samples were centrifugated at 3000 rpm for 10 min and serum samples were harvested to subpackage. After blood sampling, the heart was isolated by thoracotomy and then rinsed in saline to remove the blood. A section of the cardiac tissues were fixed in 4% neutral methanol buffer solution, whereas the rest was immediately cryopreserved in liquid nitrogen for further use. The serum and fixed tissue samples were rapidly preserved in a deep freezer at −80 °C until further use.

### Van Gieson staining

Paraffin tissue blocks were routinely dewaxed to water, and then exposed to the following reagents for processing: weight's iron haematoxylin solution for 5 min, water-washing; ascites washing for several seconds, water-washing for 5 min; Van Gieson staining solution for 3 min, removal of the staining solution; 95% alcohol for rapid separation for several seconds, anhydrous ethanol for dehydration, xylene for transparency, and neutral balsam mounting medium for sealing. Finally, collagen deposition was observed under an optical microscope.

### TUNEL fluorescence staining

Paraffin tissue blocks were dewaxed to water and then incubated with protease K in an incubator at 37 °C for 30 min, followed by addition of permeabilization wash buffer at room temperature for 20 min. Next, another incubation was performed with the reaction solution (TdT/dUTP, 1:9) at 37 °C for 2 h, followed by DAPI staining away from light for 10 min. Notably, PBS-washing (PH 7.4) was conducted for 5 min following each incubation. The tissue sections were then dried and sealed by antifade mounting medium. Microscopically, the normal nucleus was in blue in colour, whereas the positive apoptotic cells were green in colour. The apoptotic rate was measured by Image J software using the following formula: apoptotic rate = (number of apoptotic cells/total number of cells)×100%.

### ELISA for metabolites of myocardial collagen

Standard and test wells were respectively set and 100 μL of the corresponding samples were added, followed by incubation at 37 °C for 2 h. After fluid removal and well drying, biotin-antibody working solution (100 μL) was added and samples were incubated at 37 for 1 h. Next, the solution was replaced with HRP-avidin working solution (100 μL) and incubated at 37 °C for 1 h. Subsequentially, the wells were washed for five times after removing the solution, and then substrate solution (90 μL) was added for colour development away from light at 37 °C for 30 min. Finally, the termination solution (50 μL) was added and the optical density (OD) values (450 nm) were read by a microplate reader 5 min after terminating the reaction. The concentrations of serum PICP, PIIINP, and CTX-I were then calculated.

### Western blot analysis for type I/III myocardial proteins and apoptosis-related proteins

Myocardial tissues were first ground, followed by addition of protein lysis buffer, loading to a ultrasonic disintegrator, and lysis on ice. The protein products were then centrifuged and the supernatant was harvested. Protein concentration was determined using the BCA method. Next, the samples were resolved by sodium dodecyl sulphate polyacrylamide gel electrophoresis (SDS-PAGE) at a constant voltage and then transferred to polyvinylidene fluoride (PVDF) membranes at a constant voltage. After blocking non-specific binding in 5% skim milk at room temperature, membranes were incubated with primary antibodies overnight at 4 °C: anti-collagen I (1:1000), anti-collagen III (1:500), anti-Bcl-2 (1:1000), anti-Bax (1:1000), anti-caspase-3 (1:1000), and anti-β-actin (1:5000). On the next day, membranes were washed with and incubated with HRP-conjugated secondary antibody (1:5000) at 37 °C for 2 h. The ECL kit was employed for colour development to capture images. Image Lab was run to measure the grey value of each protein band, which was then used to calculate the expression of target proteins standardised by the grey value of β-actin (target protein/β-actin).

### Statistical analyses

All statistical analyses were performed using SPSS 11.5 software. Data were expressed as mean ± standard deviation ($\bar{x}\;$± s). One-way analysis of variance (ANOVA) or LSD test were used to compare differences among groups. *P* < 0.05 was considered to be statistically significant.

## Results

### Effect of QSYQ on myocardial collagen

**Figure 1.**
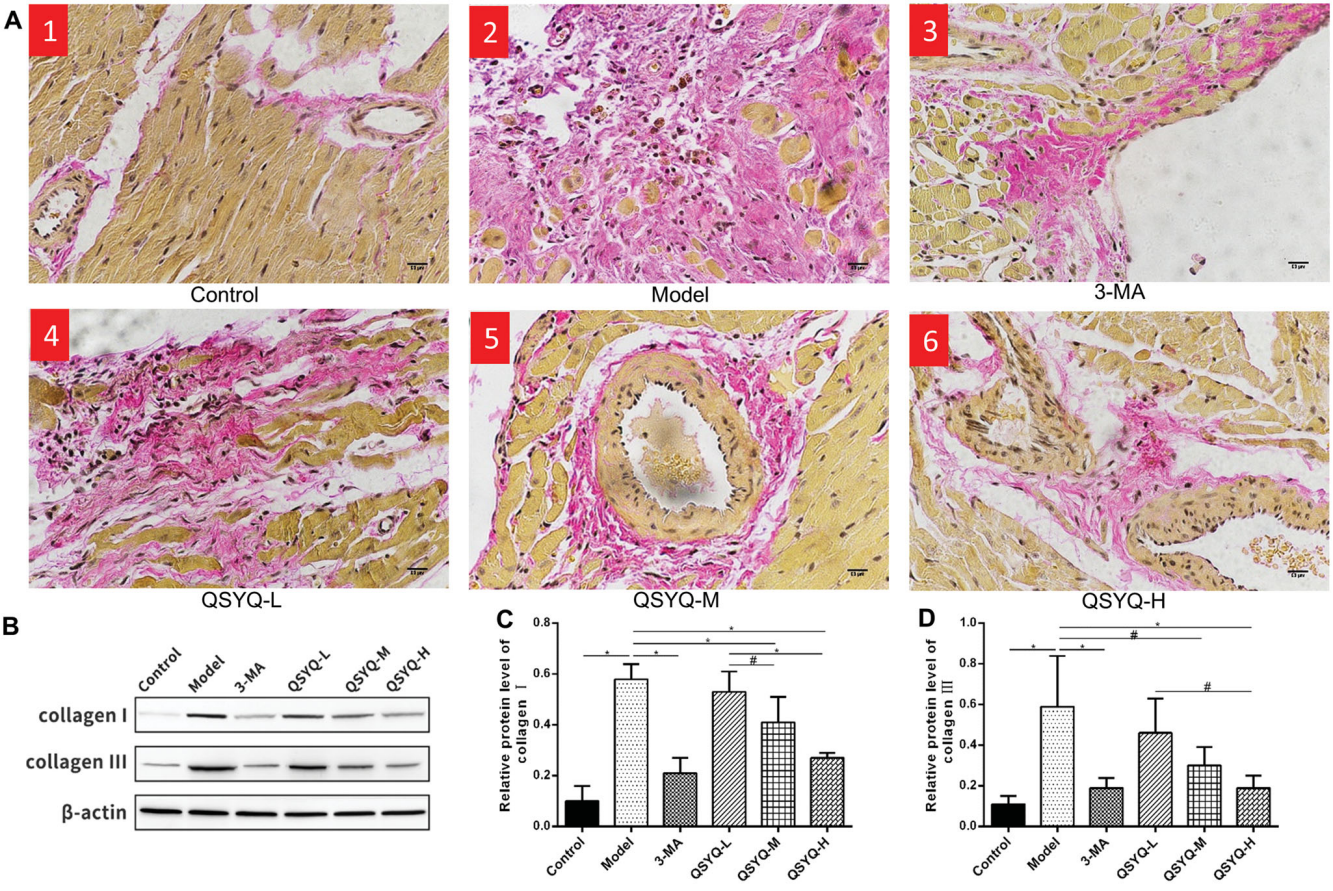
Effect of QSYQ on myocardial collagen in rats. (A) Representative pictures of myocardial VG staining (×400). (B) Western blot analysis of protein in the myocardium of rats. (C) The relative protein level of type I collagen in the myocardium. (D) The relative protein level of type III collagen in the myocardium. *p < 0.01, ^#^*p* < 0.05.

Van Gieson staining showed a small number of red myocardial tissue fibres in the control group and there was no change in myocardial interstitial fibrosis. The model group showed a lot of red myocardial tissue fibres, along with more myocardial interstitial collagen deposition in disordered arrangement. In addition, a significant decrease of red myocardial tissue fibres was observed in the presence of 3-methyladenine and QSYQ compared to the model group, suggesting that QSYQ could relieve myocardial fibrosis (Figure 1(A)).

Western blot analysis demonstrated a significant increase of type I/III myocardial collagen in the model group compared to the control (*p <* 0.01), indicating increased myocardial collagen content. The protein expression of type I/III myocardial collagens in the 3-methyladenine and QSYQ groups was reversely reduced compared to the model group (*p <* 0.01 or *p <* 0.05), suggesting that QSYQ could reduce the expression of myocardial collagen and the high-dose effect was more significant (Figure 1(B–D)).

### Effect of QSYQ compound on myocardial collagen metabolism

**Figure 2.**
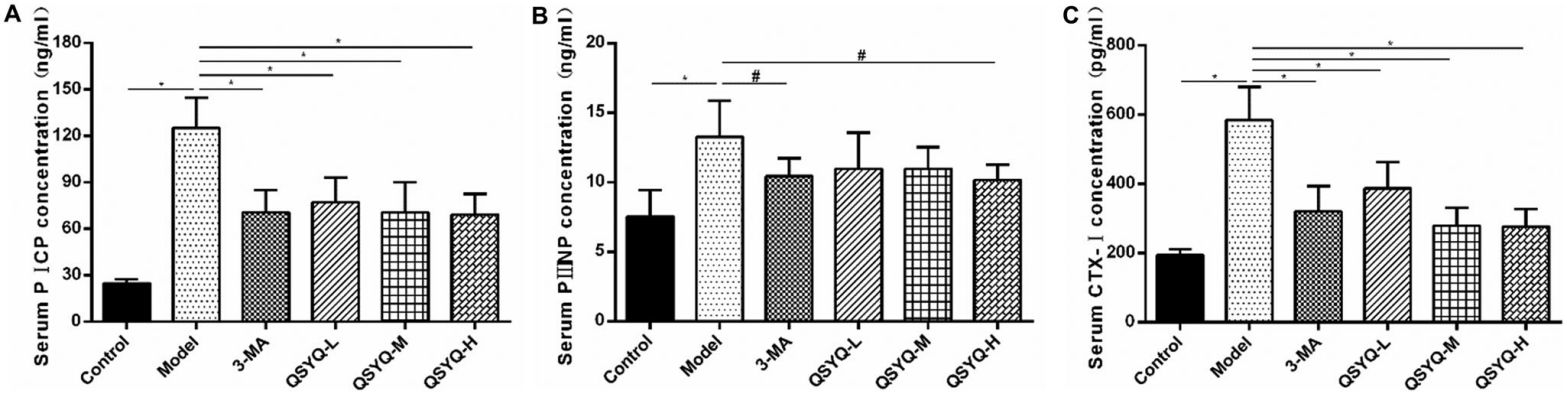
Effect of QSYQ on myocardial collagen metabolism in rats. (A) The level of serum PICP in rats (ng/ml). (B) The level of serum PIIIN in rats (ng/ml). (C) The level of serum CTX-I in rats (pg/ml). *p < 0.01, ^#^*p* < 0.05.

The concentrations of serum PICP, PIIINP and CTX-I in the model group were all higher than those in the control group (*p <* 0.01), indicating activated synthesis and degradation of myocardial collagens. However, the indexes were decreased in 3-methyladenine and QSYQ groups compared to the model group (*p <* 0.01 or *p <* 0.05), which suggested that QSYQ could effectively reduce the synthesis and degradation rate of myocardial collagen, and the effect was more significant at higher doses (Figure 2).

### Effect of QSYQ on myocardial apoptosis

**Figure 3.**
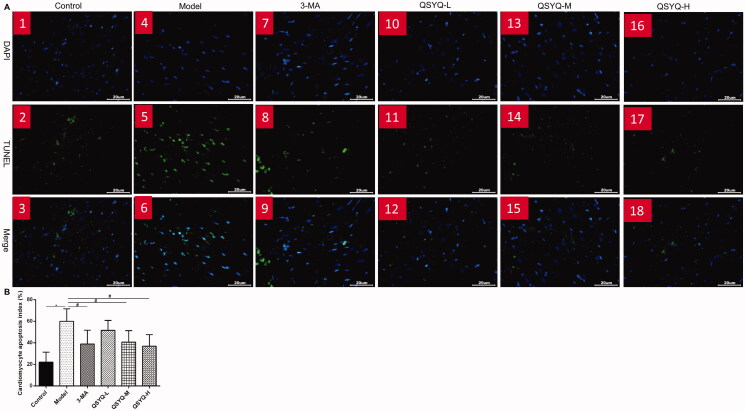
Effect of QSYQ on myocardial apoptosis in rats. (A) Typical pictures of myocardial TUNEL fluorescence staining (×400). (B) Apoptosis index of rat cardiomyocytes. *p < 0.01, ^#^*p* < 0.05.

According to fluorescence microscopy (Olympus BX51), DAPI stained nuclei showed blue fluorescence, whereas apoptotic cells were green after TUNEL staining. The myocardial cell apoptosis was significantly augmented in the model group along with a higher apoptotic rate compared to the control group (*p <* 0.01). Moreover, there was a decrease of myocardial cell apoptosis and apoptotic rate in the 3-methyladenine group and QSYQ groups compared to the control group (*p <* 0.05), suggesting that QSYQ could inhibit myocardial cell apoptosis, and the effect was more significant at a higher dose (Figure 3).

### Effect of QSYQ on myocardial apoptosis-related proteins

**Figure 4.**
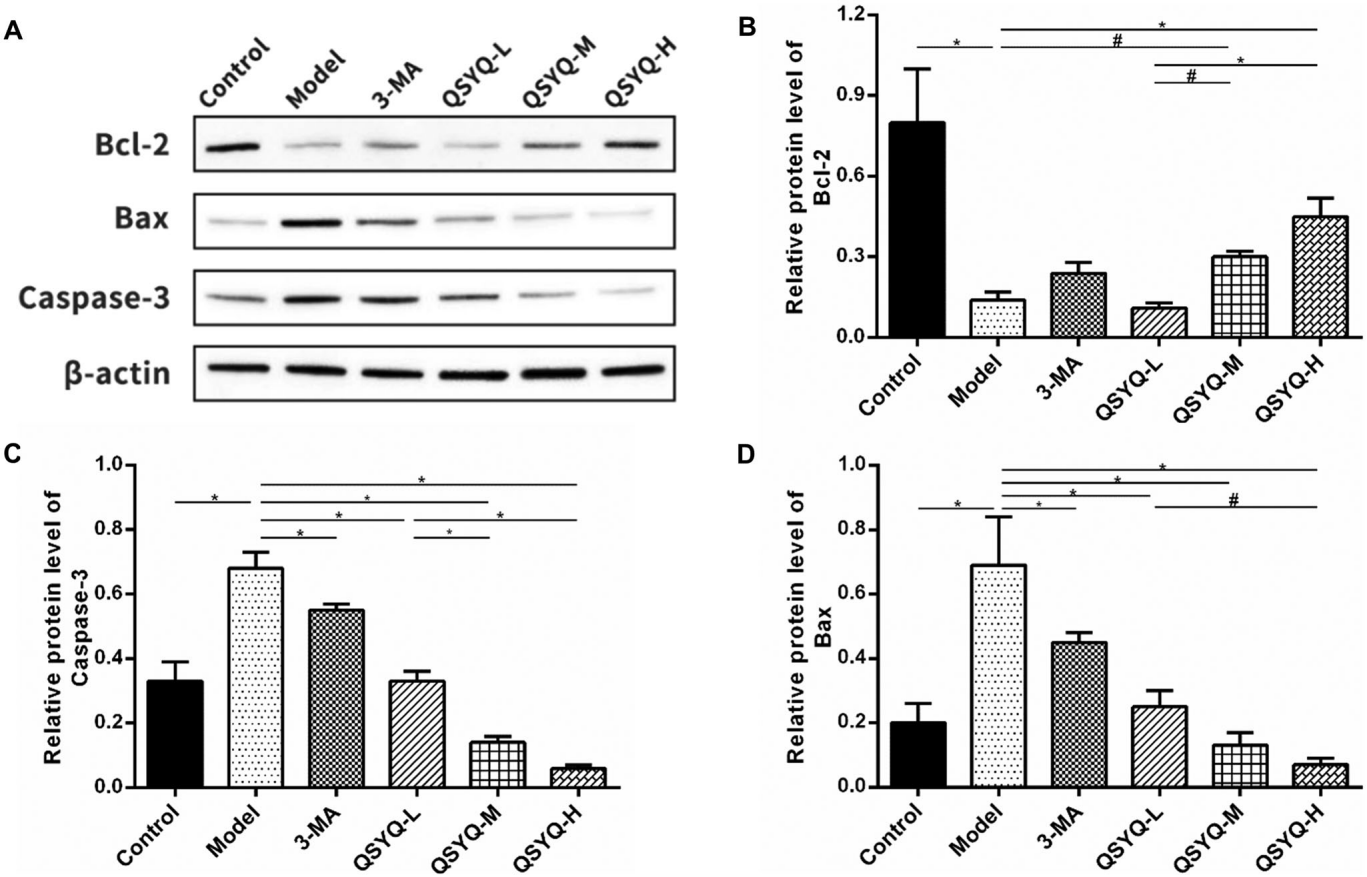
Effect of QSYQ on myocardial apoptosis related proteins in rats. (A) Western blot analysis of protein in the myocardium of rats. (B) The relative protein level of Bcl-2 in the myocardium. (C) The relative protein level of Capase-3 in the myocardium. (D) The relative protein level of Bax in the myocardium. *p < 0.01, ^#^*p* < 0.05.

The content of Bcl-2 protein was decreased (*p <* 0.01), whereas those of Bax and caspase-3 proteins were increased (*p <* 0.01) in the model group, compared to the control group. Meantime, Bcl-2 protein displayed an up-ward trend whereas Bax and caspase-3 proteins showed a down-ward trend in both the 3-methyladenine group (*p >* 0.05 or *p <* 0.01) and the QSYQ groups (*p <* 0.01 or *p <* 0.05) when compared to the model group, with the QSYQ high-dose group having a more obvious effect (Figure 4).

## Discussion

The myocardium is made up of myocardial cells and extracellular matrix (ECM) whose major fibrous protein is collagen, and mainly includes type I/III which accounts for more than 90% (Yue-Chun et al. 2016). Type I collagen is stiffer to maintain the anti-traction property of ventricular walls, whereas type III collagen is more elastic to maintain the wall’s extensibility. It is worth mentioning that the balance of quantity and proportion between type I and III collagens is crucial to the normal cardiac structure and functions. Li et al. (2019) conducted experiments in a rat model of autoimmune myocarditis, and found that excessive collagen deposition and type I/III collagen ratio increased significantly, the left ventricular diastolic function was impaired, accompanied by increased cardiac volume and weakened elasticity and contractility function. Although moderate collagen production is a repair process under certain conditions, once the balance between collagen synthesis and degradation is broken, it results in the deposition of ECM and further deposition of collagen fibres in the interstitial and perivascular areas (Lv et al. 2017). The common pathological basis of myocarditis and DCM is ECM hyperplasia, and excessive collagen deposition is an important factor for the development of myocarditis to DCM. Therefore, inhibiting collagen deposition can delay the evolution of myocarditis to DCM (Rutschow et al. 2010; Westermann et al. 2010; Xie et al. 2013; Pan et al. 2014). PICP, PIIINP, and CTX-I are myocardial collagen metabolites that are released into blood during collagen metabolism and can be detected by serology. PICP and PIIINP are the precursor polypeptides of type I/III myocardial collagen as well as biomarkers that indirectly reflect collagen synthesis in a concentration-dependent manner. On the other hand, CTX-I, as a specific marker of type I collagen degradation, indicates the rate of collagen degradation (Barasch et al. 2011; López et al. 2015). Herein, results showed that 3-methyladenine and QSYQ decreased myocardial collagen fibres and downregulated the expression of type I/III myocardial collagen and the concentrations of serum PICP, PIIINP, and CTX-I. Notably, the effect of high-dose QSYQ was more significant. Collectively, these results suggest that QSYQ could effectively lower the expression of myocardial collagen protein, and suppress the synthesis and degradation rates of myocardial collagen to reduce collagen deposition, which indicates its anti-myocardial fibrosis effect.

Apoptosis, a kind of programmed cell death has implications in multiple physiological and pathological processes. Dong et al. (2019) revealed that apoptosis is the main feature of myocardial injury, which indicates its vital role in cardiomyopathy, heart failure, myocardial infarction, and other cardiovascular diseases. In theory, myocardial cells cannot proliferate and their apoptosis leads to increased ECM compensation, that is, apoptosis initiates and participates in the process of myocardial fibrosis and heart failure induced by excessive deposition of ECM. Previous studies have reported that during the development of DCM, stimulated myocardial cells trigger endoplasmic reticulum stress to induce apoptosis, with a progressive increase in the rate and number of apoptosis (Castillero et al. 2015; Zeng et al. 2016; Sinagra et al. 2019)_._ It should be noted that myocardial cell apoptosis is the main cause of the deterioration of left ventricular function, and inhibiting the apoptosis is conducive to improving myocardial injury and restraining DCM progression. Apoptosis can be realised by both extrinsic and intrinsic pathways in a process that mainly involves the Bcl-2 and caspase family. The extrinsic pathway, also known as the death receptor pathway, takes tumour necrosis factor (TNF) receptor family as the membrane receptor of apoptosis. On the other hand, the intrinsic pathway is the mitochondrial pathway, in which the Bcl-2 family acts as a leading player. Bcl-2 is a protein that suppresses apoptosis, whereas Bax promotes apoptosis. Studies have revealed that Bcl-2 family members interact with each other to regulate apoptosis (Peña-Blanco and García-Sáez 2018; Xu et al. 2019). Both pathways can activate the caspase family, accompanied by a series of cascade reactions which enhance the apoptotic signal and accelerate cell death. According to their different roles, the caspase family members are inflammation mediators, promoters, and executioners, whereas caspase-3 activation is the core link during the apoptotic process (Vasilikos et al. 2017). Autophagy is a bidirectional-regulated process that helps to maintain stability of the internal environment. There are multiple regulatory proteins with the same functions in autophagy and apoptosis. 3-Methyladenine is a common autophagy inhibitor that suppresses the formation of autophagy by inhibiting class III phosphatidylinositol 3-kinase (class III PI3K) (Wu et al. 2010). Results obtained in this study showed that apoptosis of cardiomyocytes was evidently augmented in the model group along with a significantly increased apoptotic rate (*p <* 0.01). Moreover, the level of Bcl-2 protein was significantly decreased, Bax and caspase-3 proteins were elevated, and the Bcl-2/Bax ratio was decreased in the model group compared to the control group. Altogether, these results indicated active apoptosis of myocardial cells in rats with autoimmune cardiomyopathy. Administration of 3-methyladenine and QSYQ reduced the apoptotic rate of cardiomyocytes (*p <* 0.05), elevated Bcl-2 protein, and reduced Bax and caspase-3 proteins. These results suggest that QSYQ reduces cardiomyocytes apoptosis, especially at a higher dose.

To date, the pathogenesis of autoimmune cardiomyopathy has not yet been fully elucidated, and there is no elaborate treatment strategy. Therefore, this calls for studies to develop new therapeutic drugs for autoimmune cardiomyopathy. Traditional Chinese herbal compounds play a significant role in comprehensive intervention in a multi-component, multi-target, and multi-factorial process. The findings of this study suggest QSYQ, a traditional Chinese herbal medicine, can provide a potential therapeutic option for autoimmune cardiomyopathy. However, further studies should be conducted to elucidate the underlying mechanism of action.

## Conclusions

This study has shown that QSYQ can improve myocardial collagen metabolism, including a significant decrease of red myocardial tissue fibres, expressing type I/III collagen, and decreasing the concentrations of serum PICP, PIIINP, and CTX-I. The underlying mechanism of QSYQ action may be associated with the suppression of myocardial cell apoptosis.
